# Functionality of sugars and sugar replacers in model frozen dessert systems

**DOI:** 10.1016/j.crfs.2025.101128

**Published:** 2025-06-27

**Authors:** Qi Wang, Guido Sala, Elke Scholten

**Affiliations:** Physics and Physical Chemistry of Foods, Wageningen University, Bornse Weilanden 9, 6708, WG Wageningen, the Netherlands

**Keywords:** Effective hydration number, Ice content, Serum phase viscosity, Melting rate, Frozen dessert, Anti-freeze agents

## Abstract

Reducing sugar in frozen desserts is challenging because sugar plays a crucial role in controlling ice formation, texture, and melting behavior. Understanding how different anti-freeze agents influence these properties is essential for optimizing formulations, particularly in sugar-reduced products. We studied the effects of concentration and molecular weight of various anti-freeze agents on the ice content, ice curve, and physical properties of frozen desserts. We present a theoretical description of the ice curves of frozen solutions prepared with different anti-freeze agents. The ice content was determined by differential scanning calorimetry, from which the effective hydration number was extracted. Below 0 °C, the effective hydration number was negatively correlated with concentration and positively with molecular weight. In addition, we also determined various physical properties of the studied systems, including viscosity of the serum phase, hardness and melting properties. Hardness was related to both ice content and viscosity. However, melting parameters did not depend on both. Melting time was prolonged by high ice content, while lag time was more affected by viscosity, as higher-viscosity serum phases slowed heat transfer and delayed the onset of melting. This study shows that it is necessary to consider the characteristics of the solutes when optimizing sugar-reduced frozen dessert formulations.

## Highlights

• A theoretical model combined with DSC enabled the estimation of hydration numbers under frozen conditions.
• Hydration numbers decreased with temperature and increased with molecular weight.
• Higher ice content provided harder samples with longer melting time.
• High serum phase viscosity led by high molecular weight anti-freeze agents delayed melting onset, but not the melting rate.
• Serum phase viscosity influenced hardness up to a threshold, beyond which further increase had limited additional effect.

## Graphical abstract

**Figure:**
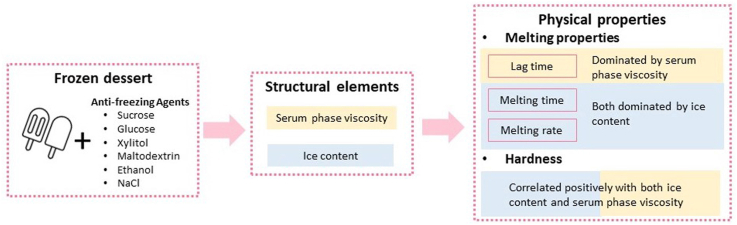

## 1 Introduction

Frozen desserts commonly contain a high concentration of sugar. The presence of sugar increases the risk of developing various chronic diseases, including obesity (Jeong et al., 2014; Park et al., 2014). Removing sugar from such products is not easy, as it plays important roles in frozen desserts: it lowers ice formation, increases serum phase viscosity and enhances air cell stabilization (McCain et al., 2018). The microstructure of frozen foods is thus strongly determined by the presence of sugar, and, therefore, its replacement in such products is challenging. Research has already shown that low-sugar frozen desserts, such as ice cream or frozen yogurt, tend to be less stable during storage (Gomes et al., 2023; Grembecka, 2015). Furthermore, the changes in physical properties induced by sugar reduction negatively affect consumer acceptance. For example, Goff and Hartel (2013) found that lowering the sugar content in ice cream resulted in lower acceptability by consumers, mainly due to increased hardness and lower creamy mouthfeel. In addition, low-sugar frozen yogurts were found to be harder and melted more slowly (Güven and Karaca, 2002; Wan et al., 2021). Therefore, sugar needs to be replaced by ingredients that are able to affect multiple properties at the same time.

As sugar limits the formation and growth of ice crystals, the freezing point of sugar-containing products decreases. The freezing point depression is related to the number of solute molecules present in the product and is mostly controlled by the size, i.e., molecular weight, of the sugar used. During partial freezing, the ice content increases as a function of temperature, which can be described by a so-called ice curve. In addition, the sugar content in the unfrozen serum phase increases as the ice content increases, which increases its viscosity (Scholten, 2014). The extent of viscosity enhancement also depends on the molecular weight of the sugars, as higher molecular weight molecules increase viscosity to a larger extent. In addition, viscosity increases with a decrease in temperature (Telis et al., 2007; Himashree et al., 2022). The properties of frozen desserts as a function of temperature, including ice content (ice curve) and serum phase viscosity, will therefore depend on the type of sugar used. Next to sugars, other food ingredients acting as anti-freeze agents are able to change the ice content and serum phase viscosity, depending on their specific molecular weight.

Besides molecular weight, the ice content in ice cream and frozen desserts also depends on the water binding capacity of the anti-freeze agents, which is related to their hydration number, i.e., the average number of water molecules interacting with one molecule of anti-freeze agents. Hydration numbers depend on temperature, concentration and molecular structure, particularly the number of hydroxyl groups that can form hydrogen bonds (Starzak et al., 2000). While the hydration numbers of sugars such as glucose and sucrose have been reported at temperatures above 0 °C (Portmann et al., 1992; Zuorro, 2021; Penkov, 2023), limited data exist for frozen conditions, where temperature effects become more significant, as shown in other studies (Gharsallaoui et al., 2008; Lerbret et al., 2005; Maneffa et al., 2017). Such information is needed to gain more insights into the precise role of sugar in frozen desserts and to design suitable sugar replacement strategies.

Hydration numbers are relevant as they concur in determining the ice curve of a specific anti-freeze agent. Therefore, they make it possible to predict part of the features of the microstructure of ice cream and frozen desserts. It is known that microstructure has a large influence on the physical properties of such foods. For example, it has been reported that in ice cream a higher ice content leads to higher hardness and iciness and lower smoothness and creaminess (Antunes et al., 2022; Muse and Hartel, 2004; Soukoulis et al., 2009). It has also been reported that changes in properties were indirectly related to changes in the ice crystal size (BahramParvar et al., 2013; Qian et al., 2022).

Besides ice content, also the serum phase viscosity is known to play a significant role in several textural properties. For example, studies have shown that higher viscosity gives “body” to ice cream, and can provide a full and creamy perception (Liu et al., 2023). In addition, viscosity can also impact perception by masking other attributes: for instance, higher viscosity has been shown to reduce iciness sensation (Amador et al., 2017) and to slow down the melting process (Wu et al., 2019; Yuennan et al., 2014). However, how viscosity and ice content relate to different melting parameters is not often discussed.

Overall, the effect of different anti-freeze agents on the structural and physical properties of frozen desserts is currently not fully understood. More specifically, it is unclear how the effect of such molecules is related to the obtained ice curve and serum phase viscosity at different temperatures. More knowledge is needed to understand how the physicochemical properties of frozen desserts are related to microstructural features. Therefore, this study aimed to investigate the effect of different anti-freeze agents on the ice curve and subsequent effects on the physical properties of frozen desserts. To limit the effect of other ingredients, we developed model systems containing only anti-freeze agents and water. Hydration numbers of sugars normally used in frozen desserts were determined at low temperatures. To investigate the separate role of ice phase and serum phase on physical properties, we also measured hardness and melting properties of frozen samples with a comparable ice content range. The studied ingredients were selected based on differences in molecular weight.

## 2 Theoretical description of the effect of anti-freeze agents on freezing point and ice curve

Ice content is an important factor in frozen desserts because it affects their texture, sensory profile, and overall quality. An excessive amount of ice can result in a dense and solid texture, while an insufficient ice content can lead to a product that melts too quickly and provides an unstable structure. Therefore, the development of ice content as a function of temperature is crucial to ensure the desired quality.

The ice content at different temperatures is determined by the type of anti-freeze agents used, and its ability to depress the freezing point of water. In this work, we derive a simplified expression for the ice curve that can be easily implemented.

In general, the freezing point depression of frozen samples (*ΔTf*) can be described as:

(1) $$\Delta Tf=Kk\cdot \frac{m_{solute}}{M_{\text{solute}}}\cdot \mathrm{i}$$

where *Kk* is the cryoscopic constant of water (−1.86 K mol^−1^ kg^−1^), *M*_*solute*_ is the molecular weight of the solute (anti-freeze agent), *m*_*solute*_ is its mass of solute and *i* its degree of dissociation. This equation is valid for dilute solutions (Leighton and Williams, 1927) and has been used in recent studies (Gallagher and Hartel, 2025; Whelan et al., 2008a). At higher concentrations, interactions between solute molecules become more significant. However, to simplify the analysis and ensure practical applicability, we assume an ideal solution, as modeling non-ideal interactions in concentrated systems is complex and requires additional parameters beyond the scope of this study. Recent studies have proposed the use of activity coefficients to more accurately predict freezing point depression and water activity in non-ideal or multi-solute food systems (Hosseini and Falamaki, 2015; Hu et al., 2022; Susrisweta et al., 2023). These approaches may particularly be more useful for modeling ice behavior in complex food matrices, where ideal solution assumptions are often inadequate. We note that the assumption of ideal behavior may lead to inaccuracies, particularly for solutes such as NaCl and ethanol, which can induce non-ideal, non-linear behavior at freezing temperatures. According to equation (1), the freezing point depression of an anti-freeze agent solution is related to the molar concentration of the solute. However, studies have demonstrated that the anti-freeze ability of small molecular solutes is influenced not only by their molar concentration, but also by their water binding ability through the formation of hydration bonds. The interaction between water and solute molecules impedes the interaction among water molecules themselves, and reduces the formation of ice (Laage et al., 2017; Roy et al., 2022; Starzak et al., 2000). Consequently, the hydration number must be taken into account when calculating the ice content of an anti-freeze agent upon decreasing temperature. In this section, we present an expression for the ice curve, which relates ice content as a function of temperature, incorporating the hydration number of the antifreeze agents. The hydration number in our study reflects not only direct solute-water interactions but also the influence of solute concentration on water availability, as derived from ice content measurements using Differential Scanning Calorimetry (DSC) and fitted with our theoretical model.

As a result of freezing, the solute becomes concentrated in the serum phase. Freeze concentration continues until the freezing point of the serum phase equals the surrounding temperature. The solute concentration, %solute, at this stage corresponds to:

(2) $$\%\text{solute}=\frac{m_{solute}}{{m_{water}+m_{solute}-m}_{\text{ice}}}$$

in which *m*_*ice*_ is the mass of ice and *m*_*water*_ is the total mass of the water.

In equilibrium, the chemical potential of the solution can be expressed as a function of the chemical potential of pure water and the amount of solute as:

(3) $$\mu_{\text{solution}}=\mu_{\text{water}}+\text{RT}\cdot \ln\left(1-x_{s}\right)$$

in which *R* is the gas constant (8.314 J⋅K^−1^⋅mol^−1^) and *x*_*s*_ is the mole fraction of the solute. This equation can be rewritten into:

(4) $$\ln\left(1-x_{s}\right)=\frac{{\Delta H}_{fus}}{R}\cdot \left(\frac{1}{T_{water}}-\frac{1}{T_{solution}}\right)$$

where ${\Delta H}_{fus}$ is the enthalpy of fusion, *T*_*water*_ is the freezing point of pure water and *T*_*solution*_ is the freezing point of the solution. This equation can be simplified to:

(5) $${x_{s}=1-e}^{a-\frac{b}{T}}$$

in which $a=\frac{{\Delta H}_{fus}}{R}\cdot \frac{1}{T_{water}}=2.646$, and $b=\frac{\Delta H}{R}=722.8$.

Due to the water binding ability of anti-freeze agents, we include an expression for the number of bound water molecules as:

(6) $$n_{bounded\;water}=\sum \left(n_{s}*h_{s}\right)$$

in which *n*_*s*_ is the mole number of solutes and *h*_*s*_ is the hydration number of the specific solute used. By combining equations (2), (5), (6), we derive an expression for the ice content as a function of temperature:

(7) $$\%ice=\frac{100\cdot {MW}_{water}}{m_{water}+m_{solute}}\cdot \left(\frac{m_{water}}{{MW}_{water}}-\left(\frac{1}{1-e^{a-\frac{b}{T}}}-1\right)\cdot \left(\frac{m_{solute}}{{MW}_{solute}}\right)-\frac{m_{solute}}{{MW}_{solute}}\cdot h_{solute}\right)$$

This equation describes the relationship between solute concentration, temperature and ice content, based on thermodynamic principles commonly used in freezing studies. While more detailed models exist—such as state diagram approaches (Vuist et al., 2022), energy balance models (López-Leiva and Hallström, 2003; Wang et al., 2025), or activity-based formulations (Colucci et al., 2020; Knopf and Alpert, 2013), our equation offers a simpler alternative. It allows for direct comparison of solutes without system-specific calibration, making it suitable for screening cryoprotectants in frozen dessert formulations.

By experimentally measuring the ice content at different temperatures, the hydration numbers of the different solutes can be obtained. In this study, the hydration numbers (*h*_*solute*_) were calculated based on equation (7) by fitting experimentally measured values of ice content at different temperatures and concentration. The derived expression is provided in the Supplementary information (Equation S(1)). We refer to *h*_*solute*_ as the effective hydration number, as it includes the results of additional non-linear effects arising in concentrated solutions, where solute clustering, altered hydrogen bond networks, and excluded volume effects modify water availability.

In this work, the effective hydration numbers for different solutes, referred to as anti-freeze agents in the remainder of the paper, was determined by measuring the ice content of different samples by DSC. It is a widely used tool for characterizing ice content and phase transitions in frozen food systems, offering both direct and indirect insights into properties in frozen conditions and melting behavior (Zhu et al., 2019; Leyva-Porras et al., 2020; Yang et al., 2022). We acknowledge that the effective hydration numbers obtained from this method may not directly be comparable to literature values measured in dilute or high-temperature systems due to non-linearity effects. For the present study, we selected glucose, sucrose, maltodextrin, ethanol, xylitol, and NaCl as anti-freeze agents. This selection allowed us to investigate the effect of the chemical structure on effective hydration numbers and ice curves. NaCl was included as representative for small, ionic solutes with strong freezing point depression but minimal impact on viscosity.

## 3 Materials and methods

### 3.1 Materials

D-(+)-glucose, sucrose, maltodextrin (with a dextrose equivalent of 16.5–19.5), xylitol and NaCl were purchased from Sigma Aldrich (Zwijndrecht, the Netherlands), and ethanol was purchased from VWR chemicals (France). Ultrapure water was purified by a Millipore Milli-Q system (Darmstadt, Germany) and was used to prepare all samples.

### 3.2 Sample preparation for ice content measurement

All solutes were dissolved in water upon stirring at room temperature for 30 min. The different samples with the tested concentrations are given in Table 1. All samples were stored at 4 °C overnight before measurement.

**Table 1:** Overview of used anti-freeze agents with respective molecular weight and tested concentrations for effective hydration number measurement.

	Mw (g/mol)	Concentration (%)
Glucose	180	15
		20
		25
Sucrose	342	15
		20
		25
Maltodextrin	800	15
		20
		25
		30
Ethanol	46.5	5
		10
		12.5
Xylitol	152	15
		20
		25
NaCl	58.4	3.0
		5.0
		7.0
		8.0

### 3.3 Ice content determination and effective hydration number (HN) calculation

The ice content of the different samples at different temperatures was measured with Differential Scanning Calorimetry (DSC) (Discovery DSC25, TA instruments, New Castle, USA). Approximately 11 mg of the sample was added to a Tzero pan and covered with a Tzero hermetic lid. Around 23 mg of aluminum was used as a reference sample and Milli-Q water was used as a blank. The samples were first cooled to −80 °C at a rate of 10 °C/min and kept constant for 1 min to initiate the crystallization of water into ice. Thereafter, the temperature was increased at a rate of 5 °C/min to different temperatures ranging between −18 °C and −2 °C with intervals of 2 °C to partly melt the ice crystals again and these temperatures were kept constant for 30 min. After 30 min, the frozen samples with a specific ice content were heated again to room temperature at a heating rate of 1 °C/min to melt the ice crystals. During this heating step, the heat flow was measured, and the measured melting enthalpy was converted to an ice content, by dividing the measured enthalpy by the enthalpy of pure ice (334 J/g). Based on the DSC results, equation (7) was used to extract the effective hydration number for different temperatures and concentrations.

### 3.4 Sample preparation for the measurement of the physical properties of the frozen desserts

Concentrations were selected after initial DSC measurements on trial samples. Based on the measured ice contents, final concentrations were chosen to span a target range of 40–70 % ice content for each anti-freeze agent. Table 2 provides an overview of the concentrations used for the different anti-freeze agents. Anti-freeze agents were dissolved in water at room temperature separately and stored at 4 °C before measurement. Maltodextrin was excluded from this sample set, as samples were too viscous.

**Table 2:** Concentrations of anti-freeze agents used to prepare frozen desserts model systems with similar ice content ranges for the measurement of physical properties.

Sample	Mw (g/mol)	Concentration (%)	Ice content at −18 °C (%)
Glucose	180	11.5	73 ± 1.2
		18	57 ± 1.0
		23	47 ± 2.2
		28	38 ± 0.9
Sucrose	342	15	70 ± 0.4
		22	57 ± 1.4
		26	49 ± 1.2
		31	42 ± 0.4
Ethanol	46.5	5	69 ± 0.8
		7.5	57 ± 0.2
		10	49 ± 0.2
		12.5	39 ± 0.8
Xylitol	152	13	70 ± 0.2
		19	59 ± 0.9
		25	51 ± 0.6
		28	41 ± 0.5
NaCl	58.4	3	73 ± 0.9
		5	62 ± 0.6
		7	50 ± 0.1
		8	42 ± 0.1

### 3.5 Viscosity of premix solutions and serum phases

Viscosity was measured with a stress-controlled rheometer (MCR301, Anton Paar, Graz, Austria) equipped with a concentric cylinder (CC) geometry (C-CC17/Ti). The viscosity of the premix solutions was measured at 5 °C. Approximately 4.7 mL sample was added to the cup and equilibrated for 1 min before each measurement. Viscosity was measured at a constant shear rate of 50 s^−1^ for 450 s. Measurements were done in triplicate to obtain average values and standard deviation.

The viscosity of the (frozen) serum phase could not be measured directly, as ice formation in the samples would affect the viscosity measurement. Therefore, to estimate the serum phase viscosity at −18 °C, a simulated serum phase was made based on the expected concentration of anti-freeze agents in the serum phase, taking into account the ice content obtained from DSC measurements. The unfrozen water (UFW) content was calculated as difference between moisture content and ice content. Table 3 presents a summary of the concentrations used in preparing the simulated serum phases for viscosity measurement. The simulated serum phases contained a higher concentration of anti-freeze agents, and, therefore, no ice formation was obtained anymore.

**Table 3:** Concentrations of anti-freeze agents used to prepare the simulated serum phase.

Sample	Concentration in the premix (%)	Concentration in serum phase (%)
Glucose	11.5	43
	18	42
	23	43
	28	45
Sucrose	15	50
	22	51
	26	51
	31	53
Ethanol	5	16
	7.5	17
	10	20
	12.5	20
Xylitol	13	43
	19	46
	25	51
	28	47
NaCl	3	11
	5	13
	7	14
	8	14

The simulated serum phase samples were stored at 4 °C before measurements were performed. Approximately 4.7 mL sample was added to the cup and viscosity was measured at a shear rate of 50 s-1, from 0 to −18 °C, with a cooling rate of 0.5 °C/min. Although no ice crystal formation was expected, some samples still exhibited minor ice crystal growth, which influenced the measurements at low temperatures. When viscosity at −18 °C could not be measured, the results obtained at lower temperatures were further extrapolated to estimate the viscosity at −18 °C. Measurements were done in triplicate to obtain average values and standard deviation.

### 3.6 Hardness of frozen samples

After freezing at −18 °C for 24 h, the hardness of samples was measured with a Texture Analyzer (TA-TX plus, Stable Micro Systems, Godalming, UK) equipped with a 50 kg load cell and a temperature control chamber. Liquid nitrogen was used to cool the chamber down to −18 °C.

All samples were frozen in a 30 mL cylinder rubber mold (φ = 65 mm, h = 9 mm) and stored at −18 °C for 24 h. Before measurements, the sample was taken out of the mold and transferred into the temperature control chamber within 30 s to minimize the effects of temperature fluctuations. During measurements, the sample was penetrated with a stainless steel cylinder probe (φ = 4 mm) up to 50 % strain and at a velocity of 1 mm/s. The maximal stress of the first peak was taken as the hardness. Hardness is expressed in MPa, calculated as the peak stress (force per area) during penetration, based on the surface area of the probe tip. Measurements were done in triplicate to obtain average values and standard deviation.

### 3.7 Melting behavior of frozen samples

**Fig. 1:**
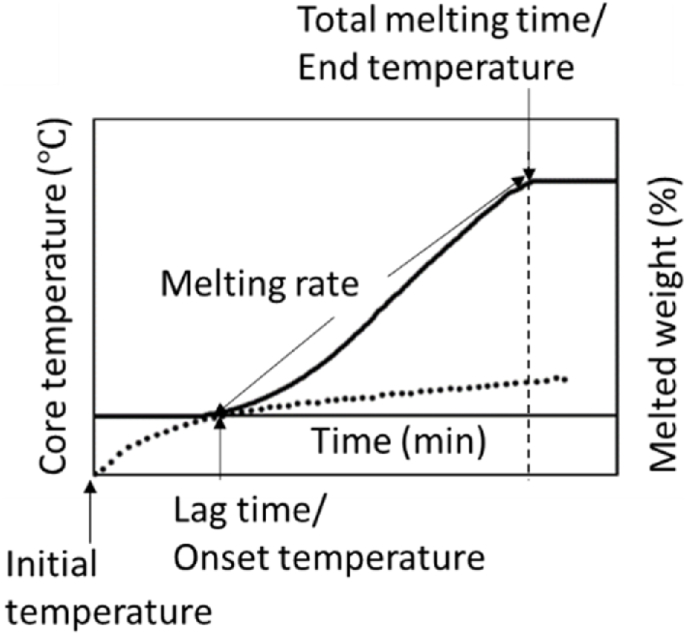
Schematic representation of a melting curve with corresponding melting parameters. The solid line represents the melted weight and the dotted line represents the core temperature of the sample.

To characterize the melting behavior of the studied model systems, 60 mL of the sample was frozen into plastic cylinders (φ = 65 mm, h = 18 mm) and stored at −18 °C for at least 12 h before measurement. The measurement was run at room temperature. The sample was placed on a 136 × 136 mm metal mesh with 10 × 10 mm holes at a total area ratio of 0.78, situated above a balance. A thermocouple was placed in the center of the sample to measure its core temperature. During melting, the dripped phase mass (g) and core temperature (°C) were recorded every 10 s until the sample was completely melted. From the melting curve (Fig. 1), different parameters were obtained: (i) total melting time, which was recorded as the time from the beginning until the time the last droplet dripped down; (ii) the lag time, which was defined as the time when the first droplet dripped down (Wu et al., 2019), and (iii) melting rate, for which the slope of the fast melting phase was used. In addition, two additional parameters were obtained: (iv) onset temperature, defined as the temperature of the first drop (at the lag time), and (v) rate of temperature increase, which was calculated by dividing the difference in the initial and end temperatures by the total melting time.

### 3.8 Statistical analysis

All measurements were performed in triplicate. Data were analyzed using SPSS software (version 25.0, IBM Corporation). Analysis of variance (ANOVA) was employed to compare the means with Duncan's test at a 5 % significance level. Linear regression analysis was conducted to evaluate the relationship between lag time and onset temperature. Coefficient of determination (R^2^) values were calculated using SPSS (version 25.0, IBM, USA) to assess model fit.

## 4 Results and discussion

### 4.1 Ice content measurement

**Fig. 2:**
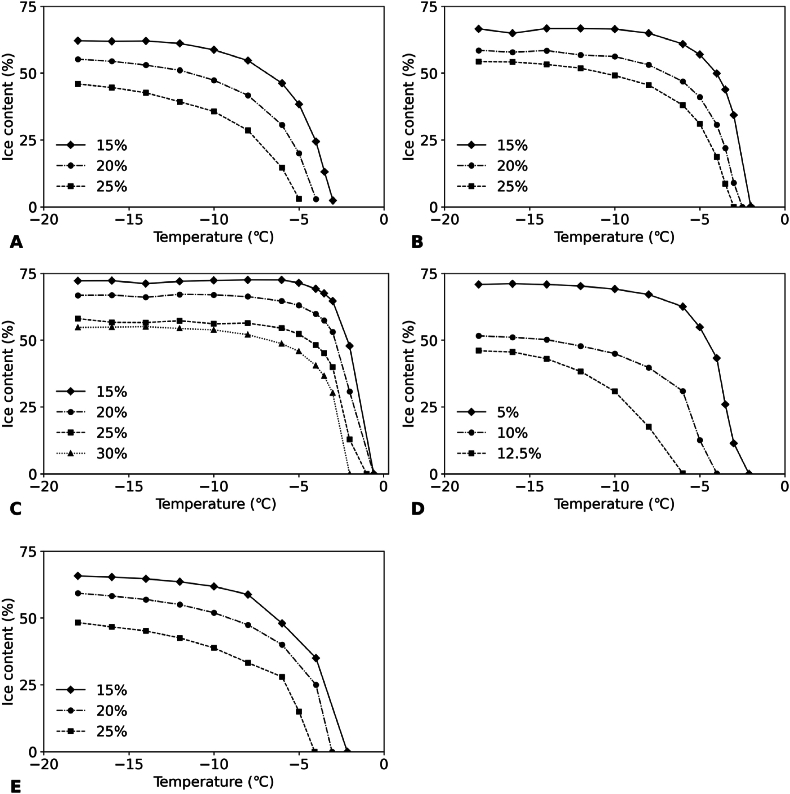
Ice content of solutions of different concentrations of anti-freeze agents as a function of temperature: A) glucose; B) sucrose; C) maltodextrin; D) ethanol; E) xylitol.

The melting behavior is strongly contributing to the overall quality of frozen desserts. As melting is directly related to changes in ice content with temperature, the ice curve of frozen systems is an important feature. To evaluate differences in the ice curves of different anti-freeze agents, we chose different ingredients based on their molecular weight: glucose, sucrose, maltodextrin, ethanol, xylitol and NaCl (Table 1). We then measured the ice content of solutions of these anti-freeze agents within a temperature range from −18 °C to the melting point of the specific sample. The results for the different anti-freeze agents are shown in Fig. 2. Unfortunately, no consistent results could be obtained for NaCl. Due to the crystallization of NaCl, the change in enthalpy due to ice formation could not be determined from the DSC heat flow curve. Therefore, no results for NaCl are included in the figure. To illustrate this limitation, Figure S1 (Supplementary information) presents a DSC curve of a 7 % NaCl sample showing crystallization behavior, which overlapped with ice transitions and prevented reliable enthalpy quantification during melting.

Obviously, the ice content decreased with increasing temperature and concentration of anti-freeze agent (Fig. 2). Among the five agents, maltodextrin had the weakest anti-freeze ability (Fig. 2C): the studied maltodextrin had the highest molecular weight (800 g/mol), and its solutions had therefore the lowest molality. In contrast, ethanol, with the lowest molecular weight (46 g/mol), showed the strongest effect of freezing and already lowered the ice content at lower concentrations. This observation is consistent with the colligative nature of freezing point depression, which has been shown by other authors (Lamas et al., 2022; Wu et al., 2023). We also observed that the shape of the ice curve varied among the different anti-freeze agents. So, for a similar ice content at temperatures in the range between −15 and −18 °C, the melting point (where the ice curve crosses the y-axis) could still be different. For example, in the case of sucrose (Fig. 2B), the melting point was approximately −3 °C when the ice content at −18 °C was 75 %. However, at the same ice content at −18 °C, the melting point of maltodextrin (Fig. 2C) was only around −1 °C. So, the molecular weight also had an influence on the evolution of the ice content over time, which may play an important role in the melting process. The ice curves of xylitol (Fig. 2E) were the most similar to those of glucose (Fig. 2A), likely due to their similar molecular weights.

### 4.2 Effective hydration numbers of anti-freeze agents

From the ice curves shown in Fig. 2, the effective hydration numbers of the different anti-freeze agents were calculated using Equation (7). We point out that the effective hydration number accounts for interaction effects arising in non-dilute conditions, where increased solute concentration leads to competition for water molecules, altered hydrogen bonding, and changes in water mobility. These effects are reflected in the effective hydration number, as derived from ice content measurements using DSC and fitted with our theoretical model. If the effective hydration number were independent of temperature, then one single effective hydration number could be used to fit the experimental data with Equation (7). However, in all cases, we found that this was not possible, confirming that the effective hydration number of the different anti-freeze agents was temperature-dependent. Therefore, we extracted the effective hydration numbers at different temperatures to gain insight into their temperature dependency. In addition, we also determined the effective hydration numbers of solutions with different anti-freeze agent concentrations. The results are shown in Table 4.

**Table 4:** Effective hydration numbers of the studied anti-freeze agents at different concentrations and temperatures.

	Mw (g/mol)	Concentration (%)	Effective hydration number					
			−18 °C	−16 °C	−14 °C	−12 °C	−10 °C	−8 °C
Glucose	180	15	10.5	9.8	9.4	9.8	10.8	13.8
		20	4.9	4.6	4.6	4.5	4.6	5.7
		25	5.0	4.9	4.9	5.0	5.2	6.1
Sucrose	342	15	17.4	19.9	15.8	14.9	13.8	13.4
		20	14.9	14.8	13.6	13.4	12.7	13.1
		25	10.9	10.3	9.8	9.7	9.9	10.2
Maltodextrin	800	15	33.1	32.2	34.5	30.8	28.1	21.9
		20	24.4	23.5	24.4	20.8	19.5	18.4
		25	25.2	28.8	26.2	23.8	24.1	21.1
		30	17.6	16.8	15.6	15.4	14.5	14.5
Ethanol	46.5	5	7.3	6.8	6.2	5.5	4.8	4.6
		10	5.1	4.6	4.3	3.8	3.4	3.1
		12.5	3.2	3.0	2.6	2.3	2.2	2.3
Xylitol	152	15	8.5	8.3	8.0	7.8	7.7	7.6
		20	6.0	6.0	6.0	6.3	6.8	6.8
		25	6.4	6.5	6.4	6.5	6.8	6.7

∗Effective hydration numbers were derived from fitting ice content data and were calculated based on averaged experimental values. Therefore, standard deviations are not provided.

Based on these results, we could confirm the link between effective hydration number and molecular weight: the effective hydration number increased with increasing molecular weight. Ethanol, with the lowest molecular weight, had an effective hydration number ranging from 2.2 to 7.3, whereas that of maltodextrin ranged from 14.5 to 34.5. The effect of the molecular weight on the effective hydration number can be explained with the higher number of contact points for interactions with water molecule. In other studies, the formation of more hydrogen bonds for larger molecules has also been shown with infrared spectroscopy (Ni et al., 2017; Shalaev et al., 2019). As expected, we found that the effective hydration numbers of glucose and sucrose at −18 °C were between those of ethanol and maltodextrin, in a range of 5.0–13.8 and 10.2 to 17.4, respectively. These values are higher than reported by others. An earlier study showed that the effective hydration number of glucose and sucrose at 20 °C was 3.45 and 6.63, respectively (Mohammed and Reiser, 1995), but also values between 1.8 and 6 have been reported for sucrose by fitting experimental data through different models. These modeling predictions consider the thermodynamic variances between low and high concentration sucrose solutions, resulting in variations in the effective hydration number (Starzak et al., 2000; Tas et al., 2022). In many studies, hydration numbers were obtained with spectrophotometry or nuclear magnetic resonance (NMR). In our study, effective hydration numbers were obtained by DSC. The advantage of DSC is that measurements can also be performed at temperatures lower than 0 °C, which is not possible with other methods. Our results were quite temperature-dependent. Therefore, it can be assumed that more accurate ice curves can be constructed when using effective hydration numbers obtained with DSC, as they reflect interactions between water and anti-freeze agents in frozen conditions. With our approach, we were able to determine the effective hydration numbers in a temperature range from −8 °C to −18 °C.

Overall, for most anti-freeze agents, the effective hydration numbers gradually increased at lower temperatures. This effect was especially clear for ethanol: at 5 %, the effective hydration number increased from 4.6 at −8 °C to 7.3 at −18 °C. The effect of temperature on effective hydration number can be explained for most samples by decreased hydrogen bond formation. As the temperature increases, molecular motion increases, hindering the formation of intermolecular bonds. Conversely, lower temperatures promoted the formation of hydrogen bonds, causing solvent and water molecules to interact more tightly (Mizan et al., 1996; Galamba, 2013), in line with other studies (Starzak et al., 2000; Gharsallaoui et al., 2008; Zuorro, 2021). However, for glucose, the temperature dependence of effective hydration number was non-monotonic. For example, at 15 % glucose, when temperature increased from −14 °C to −8 °C, the effective hydration number increased from 9.4 to 13.8, rather than decreasing as seen with other solutes. This may indicate that glucose–water hydrogen bonds are relatively weak and less responsive to further cooling, or that the structure of the hydration layer around glucose changes in a more complex manner at low temperatures. Previous studies have suggested that monosaccharides like glucose interact less strongly with water's hydrogen bond network (Suzuki, 2007; Marshall et al., 2022), which may explain this special behavior. Therefore, they may be less affected by temperature changes, which could explain why the trend of the glucose effective hydration number is different from other samples.

Although temperature changes did not lead to large variations in effective hydration numbers for some anti-freeze agents, larger effects were observed as a function of concentration. For all anti-freeze agents, the effective hydration number became smaller in high-concentration samples. For example, the effective hydration number of glucose at −18 °C decreased from 10.5 to 5.0 when the concentration increased from 15 to 25 %. For ethanol, the effective hydration number at the same temperature dropped from 7.3 to 3.2 as concentration increased from 5 to 12.5 %, a decrease of 56 %. To visualize these trends more intuitively, 3D plots were added to show how effective hydration number changes with temperature and solute concentration for each solute (Figure S2, Supplementary information). A fixed color scale was used to allow visual comparison between solutes. The plots clearly show different patterns in hydration behavior. For example, a sharp increase in effective hydration number can be observed for maltodextrin, whereas relatively flat profiles for xylitol and ethanol were obtained. These concentration-dependent trends may be related to how water interacts with the anti-freeze agents. At higher sugar concentrations, effective hydration numbers decreased, likely because water molecules preferentially bond with each other rather than with sugar. As sugar molecules become surrounded by hydration shells, excess water forms self-associated clusters, reducing solute-water interactions (Ekdawi-Sever et al., 2001; Ohtake and Wang, 2011; Penkov, 2021). Xylitol showed the lowest concentration dependence of all anti-freeze agents. The effective hydration number of xylitol was around 7, independently of concentration. This value is close to that found by Płowaś-Korus and Buchner (2019), who reported a value of 5.66 at 20 °C. The stability of xylitol's effective hydration number across concentrations suggests that its molecular interactions with water remain relatively unchanged, possibly due to the symmetry of its hydroxyl groups and the way it structures local water molecules.

To conclude, we found that the anti-freeze capacities were dependent on both the molecular weight and the effective hydration number. Effective hydration numbers were shown to be both temperature- and concentration-dependent. With these numbers, the specific ice content of anti-freeze agents solutions at different temperatures can be easily calculated and changes in ice content during melting can be predicted using equation (7). The differences between effective hydration numbers have practical implications for frozen dessert formulation. Solutes with higher effective hydration numbers, such as maltodextrin and sucrose, tend to bind more water and reduce ice formation, which may lead to softer texture. Therefore, selecting an anti-freeze agent with appropriate effective hydration behavior is crucial for designing texture, as they are expected to influence different physical properties of the frozen dessert.

### 4.3 Properties of model frozen desserts

#### 4.3.1 Physical characteristics

Next to differences in ice curves, various anti-freeze agents will also have a different effect on the viscosity of both the initial solution and the serum phase of the frozen samples. The serum phase viscosity develops while the ice content increases, and, therefore, the serum phase viscosity is also directly related to the specific ice curves of the samples. In the previous section, we showed that small molecules were more efficient in decreasing the ice content. To be able to compare the effect of the serum phase viscosity on the physical properties of frozen desserts, samples with different anti-freeze agents were made with a similar range in ice content at −18 °C, from 40 % to 70 %, by adjusting the anti-freeze agent concentration. Maltodextrin was excluded due to a higher contribution to serum phase viscosity. NaCl was included as its effect on viscosity was limited and similar to that of ethanol.

The premix viscosity of the initial mix was measured at 4 °C, and the serum phase viscosity of the simulated non-frozen serum phase was measured at −18 °C. Freezing point, ice content, premix viscosity and serum phase viscosity of the different samples are summarized in Table 5.

**Table 5:** Ice content, premix viscosity and serum phase viscosity of model frozen desserts with different anti-freeze agents.

Sample name	Concentration (%)	Freezing point (^0^C)	Ice content (%) (-18 °C)	Premix viscosity (mPa·s) (4 °C)	Serum phase viscosity (mPa·s) (−18 °C)
Glucose	11.5	−1.34	73 ± 1.2	2.3 ± 0.3	35.4 ± 0.2
	18.0	−2.27	57 ± 1.0	2.9 ± 0.1	43.4 ± 0.7
	23.0	−3.09	47 ± 2.2	3.8 ± 0.2	60.4 ± 0.4
	28.0	−4.02	38 ± 0.9	5.0 ± 0.9	72.8 ± 0.6
Sucrose	15.0	−0.96	70 ± 0.4	2.7 ± 0.5	138.9 ± 0.7
	22.0	−1.53	57 ± 1.4	3.9 ± 0.1	210.2 ± 1.3
	26.0	−1.91	49 ± 1.2	4.9 ± 0.2	216.4 ± 1.1
	31.0	−2.44	42 ± 0.4	6.5 ± 0.2	362.2 ± 1.5
Ethanol	5.0	−2.11	69 ± 0.8	1.9 ± 0.4	1.9 ± 0.1
	7.5	−3.24	57 ± 0.2		
	10.0	−4.44	49 ± 0.2		
	12.5	−5.71	39 ± 0.8		
Xylitol	15.0	−2.16	65 ± 0.2	2.5 ± 0.1	20.5 ± 0.2
	20.0	−3.06	58 ± 0.9	2.9 ± 0.4	30.9 ± 0.1
	25.0	−4.08	47 ± 0.6	3.7 ± 0.2	49.6 ± 0.3
	30.0	−5.24	32 ± 0.4	4 0.7 ± 0.3	60.6 ± 0.1
NaCl	3.0	−1.54	71 ± 0.9	2.0 ± 0.2	2.0 ± 0.4
	5.0	−2.63	61 ± 0.1		
	7.0	−3.76	50 ± 0.1		
	8.0	−4.36	42 ± 0.4		

The viscosity of the serum phase of samples made with sucrose was the highest. After freezing, the serum phase viscosity of these samples varied between 139 and 362 mPa·s, depending on concentration. Glucose and xylitol had a lower contribution to viscosity, and the effect of concentration on premix viscosity was similar. Serum phase viscosity ranged between 35 and 73 mPa·s for glucose and between 20 and 60 mPa·s for xylitol. This small difference can be explained by the higher molecular weight of glucose (180 g/mol) compared to xylitol (152 g/mol). The serum phase viscosity appeared thus also to be related to molecular weight. In line with this observation, the contribution of both ethanol and NaCl to premix and serum phase viscosity was limited due to their much lower molecular weight (46.5 and 58.4 g/mol). For ethanol and NaCl, the serum phase viscosity remained nearly constant across the tested concentrations. Due to their low overall viscosity contribution, no significant differences were detected, (Table 5).

#### 4.3.2 Hardness of the model frozen dessert

**Fig. 3:**
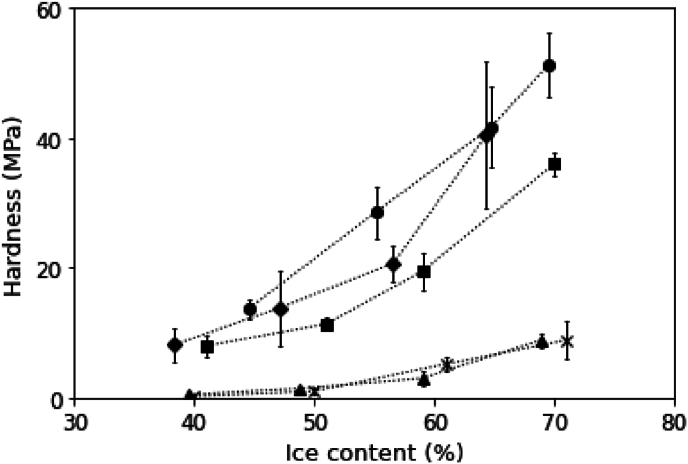
Stress (as a measure for hardness) as a function of the ice content of model frozen desserts with different anti-freeze agents. Different symbols represent different samples: ◆) glucose; ●) sucrose; ■) xylitol; ▲) ethanol; × ) NaCl. The lines are added as a guide to the eye.

Hardness is considered to be one of the most important physical properties of frozen desserts, and is related to both product stability and overall sensory impression (Muse and Hartel, 2004). Both ice content and serum phase viscosity are expected to have an influence on hardness. To ascertain this, the stress needed to penetrate the frozen samples was measured as a measure for the hardness (Fig. 3).

Naturally, hardness increased with increasing ice content (Fig. 3). For sucrose samples, hardness increased from 12.25 MPa to more than 50 MPa with increasing ice content from 40 to 70 %. Similar trends were observed for the samples with the other anti-freeze agents. Although similar trends were seen, the absolute values of the samples were very different, and we could separate the samples into two groups: a high viscosity group (sucrose, glucose and xylitol) and a low viscosity group (ethanol and NaCl), indicating that the serum phase viscosity played a large role in determining hardness. However, within the high viscosity group, limited differences in hardness were observed, even though their serum phase viscosity was different (Table 5).

These results show that besides serum phase viscosity and ice content, other factors influence the physical properties of frozen samples. Although an increase in viscosity from 2 mPa·s (ethanol and NaCl) to 43.4 mPa·s (glucose) corresponded to a large increase in hardness, an additional increase to 138 mPa ·s (sucrose) had no further effect in samples with an ice content around 70 %. Similarly, a large increase in hardness was seen for the glucose sample with a serum phase viscosity of 60.4 mPa·s (ice content = 47 %), but no further increase for the sucrose sample with a serum phase viscosity of 216 mPa·s (ice content = 49 %). So, although serum phase viscosity affects hardness, the effect of serum phase viscosity was not always clear. This may suggest that a minimum viscosity is needed to affect hardness, but after reaching such a threshold the effect of viscosity becomes less pronounced. In the lower viscosity range, we indeed observed a positive relation between viscosity and hardness. However, as viscosity exceeded a certain value, we observed no further significant increase in hardness by comparing glucose and sucrose samples.

In addition, the xylitol samples had lower hardness compared to the glucose samples, which cannot be solely explained by serum phase viscosity, as viscosity values for xylitol and glucose largely overlapped (Table 5). Serum phase viscosity therefore did not always have a direct effect on hardness. This partly contradicts the claim that serum phase viscosity directly affects hardness (Amador et al., 2017; Kurultay et al., 2010; Wu et al., 2019). One potential reason for this difference is that our model system is simplified, consisting only of water and anti-freeze agents. In the models studied by other authors, additional ingredients such as fat, proteins and stabilizers can form a more complex matrix with the serum phase. The interactions among such ingredients likely enhance the structural role of viscosity by supporting the ice network. In addition, our system did not involve overrun and fat network, factors known to affect texture properties of frozen dessert. Therefore, in our system, the absence of such structural contributors may explain why hardness was more directly governed by ice content, while the effect of viscosity appeared to plateau beyond a certain threshold.

#### 4.3.3 Melting behavior

**Fig. 4:**
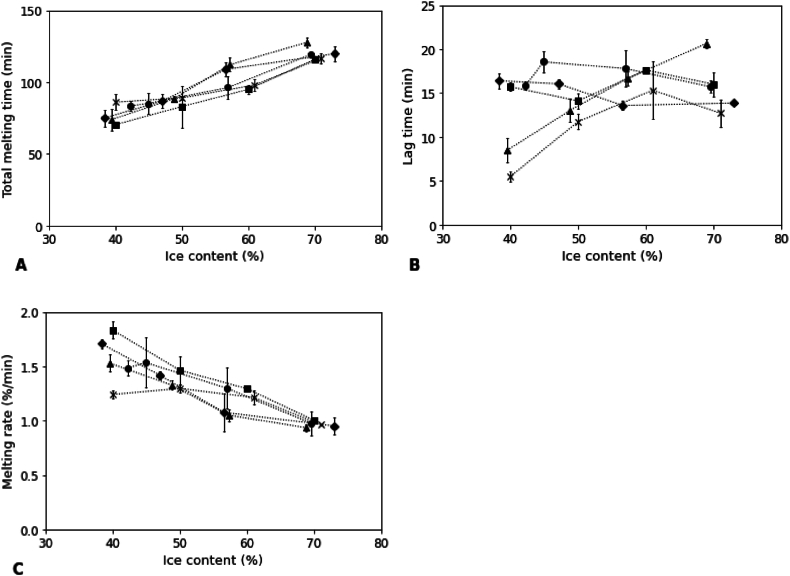
Melting parameters as a function of ice content of model frozen desserts with different anti-freeze agents: A) total melting time; B) lag time; C) melting time. Different marks represented different samples: ◆) glucose; ●) sucrose; ■) xylitol; ▲) ethanol; × ) NaCl. Lines were added as a guide to the eye.

By comparing the melting time, melting rate and lag time of the different samples, the effects of different structural characteristics of the frozen model systems on melting were investigated (Fig. 4). For all the samples, melting time increased with ice content (Fig. 4A). When ice content increased from 30 % to 70 %, the total melting time increased from 1 h to approximately 2 h. This was expected, as a higher amount of ice requires a longer time to melt completely. All samples seemed to follow the same trend, and serum phase viscosity did not appear to affect the total melting time. So, the total melting time was just determined by the total amount of ice, independently of the properties of the serum phase in which the ice crystals were embedded.

Although for all samples the total melting time followed a similar trend with increasing ice content, differences were observed at the beginning of the melting process, as the lag time depended also on the serum phase viscosity of the samples (Fig. 4B–Table 4). For samples with low viscosity, the lag time increased with increasing ice content. For the ethanol sample, the lag time increased from 8 min to 20 min when the ice content was increased from 40 % to 70 %, while for NaCl, it increased from 5 min to 15 min. For samples with higher serum phase viscosity (glucose, sucrose, and xylitol), much higher values of lag time were observed. In these cases, the lag time ranged between 13 min and 20 min, but a limited impact of the ice content was observed. As serum phase viscosity became more important, the effect of ice content was less pronounced. Thus, a certain critical viscosity probably needs to be achieved to limit the effect of the ice phase. Higher lag times are often explained by the fact that samples with a higher serum phase viscosity have more resistance to flow (Bourne, 2002; Wu et al., 2019; Schultze-Jena et al., 2020). In addition, the serum phase is claimed to provide more cohesion within the system (Goff and Hartel, 2013; Wang and Hartel, 2020; Yuennan et al., 2014). However, in our study, only minor variations in lag time were noted between sucrose and glucose samples, despite notable differences in the viscosities of their serum phases (42.8 MPa·s and 138 MPa·s). Thus, these findings suggest that lag time is not exclusively linked to serum phase viscosity, and other factors of the anti-freeze agents may also contribute. The morphology of the ice crystal network may also play a role, although we cannot make firm conclusions based on the present results. Besides serum phase viscosity, the structure of the ice crystal network can also influence lag time. A denser and more interconnected ice network may help delay the onset of melting by slowing water mobility and promoting structural integrity during heating (Tan et al., 2021). The difference between our outcomes and those of others may once more be ascribed to variations in the recipe. In more complex formulations, other ingredients such as stabilizers, fat and proteins may further enhance melt resistance by reinforcing the matrix, trapping water, and altering thermal response. These interactions were not present in our simplified model systems but are relevant in practical applications.

After reaching the lag time, the samples started to melt, and a mixture of thawed water and serum phase started to drip from the sample until all the ice was melted. We determined the melting rate values in this time frame and plotted them versus ice content (Fig. 4C). The melting rate decreased with increasing ice content. Depending on the anti-freeze agent, the melting rate decreased approximately from 1.75 %/min to 1 %/min (Fig. 4C). This points out that the melting rate is closely related to the ice content, which is logical, as melting time was also closely related to ice content. The low viscosity samples (alcohol and NaCl) showed the same pattern, indicating that viscosity had a limited effect on the melting rate, as observed for melting time. Other studies have also shown that the melting rate was mostly affected by ice content (Kawai and Hagiwara, 2018; Whelan et al., 2008b). This can be explained by the fact that melting is determined by the heat transfer through the sample. The heat transfer rate is influenced by thermal conductivity, which is mainly related to ice content, and less by other features. As ice has a higher thermal conductivity (2.2 W/m·K) than water (0.6 W/m·K) (Chuvilin et al., 2018), melting in ice cream and frozen desserts is mostly determined by the ice content. Samples with higher ice content require more energy to melt, resulting in a longer melting process.

Some studies have proposed that higher melting rates are also associated with increases in serum phase viscosity, and that the flow behavior of the mixture has a slight effect on the melting rate of ice cream (Goff and Hartel, 2013; Muse and Hartel, 2004; Yuennan et al., 2014). However, our results indicate that although the lag time was affected by serum phase viscosity, the melting process was dominated by the ice content. It is important to note that the cited studies did not take into consideration the variability in ice content among the samples, which could potentially explain the results. In addition, also air content in ice cream often varies among samples with different serum phase viscosity. As air has a large effect on heat transfer (VanWees et al., 2020), differences in melting rate among samples may also be related more to changes in overrun, instead of differences in serum phase viscosity. In our study, we are able to isolate the effect of viscosity on melting rate, as the samples were prepared without air. Since limited differences were observed among samples, our results suggest that the serum phase viscosity had only a small effect on melting rate and melting time. Thus, we concluded that in our samples melting rate and melting time were more related to ice content than to serum phase viscosity, while lag time was more affected by serum phase viscosity in low ice content samples. However, not all results can be explained by ice content and viscosity only.

#### 4.3.4 Core temperature profile during melting of the frozen dessert

**Fig. 5:**
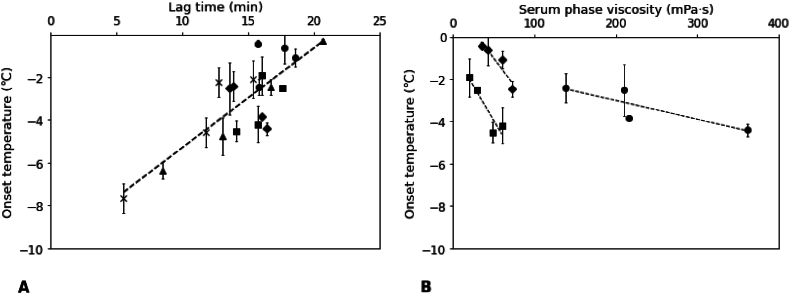
Onset temperature versus lag time (A) and serum phase viscosity (−18 °C) (B) of model frozen desserts with different anti-freeze agents. Different marks represent different samples: ◆) glucose; ●) sucrose; ■) xylitol; ▲) ethanol; × ) NaCl. The lines were added as a guide to the eye.

As discussed previously, our findings indicate that the lag time in the melting process was influenced by both the ice content and the serum phase viscosity of our samples. To gain more understanding of the melting process, we also measured the change in temperature of the samples over time. As the ice content decreased, an increase in the amount of unfrozen serum was obtained, in combination with an increase in temperature. When the lag time was delayed by the structure (serum phase viscosity) of the frozen sample, we thus expected a higher (onset) temperature at the end of the lag time. We therefore expected this onset temperature to be affected by the serum phase viscosity of the samples. In Fig. 5, the onset temperature of the studied samples is plotted versus both lag time and serum phase viscosity.

Overall, the onset temperature indeed increased when lag time increased (Fig. 5A), with a linear relation (R^2^ = 0.67). We also observed that the onset temperature was related to serum phase viscosity (at −18 °C) for the samples with glucose, sucrose, and xylitol, but their results did not overlap. For sucrose samples, the onset temperature decreased from −2 °C to −4 °C as the serum phase viscosity increased from 138 mPa·s to 362 mPa·s. For glucose, the onset temperature decreased from almost 0 to −2 °C, but for a much smaller viscosity range (<100 mPa·s). Also, xylitol samples showed a range of serum phase viscosity similar to that of glucose, but its onset temperatures ranged from −2 to −4.2 °C. From these results, it seems that with increasing serum phase viscosity, the changes in onset temperature became smaller.

A higher serum phase viscosity appeared to slow down heat transfer within the sample, effectively acting as a thermal insulator. As a result, more energy was required to induce temperature changes, delaying the overall melting process. Additionally, samples with a high serum phase viscosity have a higher resistance to flow, which could significantly affect the heat transfer due to the convection induced by a temperature gradient (Park et al., 2022; Sloman et al., 2020). This could explain the slow change in core temperature observed in samples with a high serum phase viscosity (sucrose), as the serum phase may impede heat transfer and result in a slower increase in temperature during melting. However, as the curves did not overlap, also other factors seemed to influence the melting behavior besides viscosity. This again may be more related to the ice crystal network or ice crystal size.

## 5 Conclusion

This study provides a systematic approach to understanding how different anti-freeze agents influence the microstructure and physical properties of frozen desserts. By developing a theoretical model for ice content, we demonstrated that effective hydration numbers decrease with increasing concentration and temperature, but increase with molecular weight. The ice curves obtained from DSC measurements allowed us to quantify these effects and predict ice content across different formulations. Our findings confirmed that ice content is the primary determinant of hardness and melting time, with higher ice content leading to firmer textures and longer melting durations. Serum phase viscosity influenced lag time, delaying melting onset due to reduced heat transfer, but had no significant effect on overall melting rate. Interestingly, viscosity effects were not always linear. Above a certain threshold, further increases in viscosity did not strongly impact hardness or melting behavior. These insights highlight the dual role of anti-freeze agents in structuring frozen desserts by controlling both ice phase properties and serum phase behavior. The results provide a scientific foundation for optimizing frozen dessert formulations, particularly for sugar-reduced products, by balancing molecular weight, hydration effects, and viscosity contributions. As the presented insights were obtained with a simplified model system, caution is needed when extrapolating the learnings to complex food matrices containing other ingredients.

## CRediT authorship contribution statement

**Qi Wang:** Investigation, Data curation, Methodology, Visualization, Writing – original draft. **Guido Sala:** Methodology, Conceptualization, Supervision, Writing – review & editing, Funding acquisition. **Elke Scholten:** Methodology, Conceptualization, Supervision, Writing – review & editing, Funding acquisition.

## Declaration of competing interest

The authors declare that no competing interest exists.

## Data Availability

Data will be made available on request.

## References

[bib1] Amador J., Hartel R., Rankin S. (2017). The effects of fat structures and ice cream mix viscosity on physical and sensory properties of ice cream. J. Food Sci..

[bib2] Antunes A.E.C., Gallina D.A., Rodrigues V.C. da C., da Cunha D.T., da Cruz A.G., Ranadheera C.S., Nazzaro F., Mortazavian A.M. (2022). Dairy Foods.

[bib3] BahramParvar M., Mazaheri Tehrani M., Razavi S.M.A. (2013). Effects of a novel stabilizer blend and presence of κ-carrageenan on some properties of vanilla ice cream during storage. Food Biosci..

[bib4] Bourne M.C. (2002). Texture, viscosity, and food. Food Texture Viscosity.

[bib5] Chuvilin E., Bukhanov B., Cheverev V., Grechishcheva E. (2018). Impact of Thermal Conductivity on Energy Technologies.

[bib6] Colucci D., Fissore D., Barresi A.A., Braatz R.D. (2020). A new mathematical model for monitoring the temporal evolution of the ice crystal size distribution during freezing in pharmaceutical solutions. Eur. J. Pharm. Biopharm..

[bib7] Ekdawi-Sever N.C., Conrad P.B., De Pablo J.J. (2001). Molecular simulation of sucrose solutions near the glass transition temperature. J. Phys. Chem. A.

[bib8] Galamba N. (2013). Water's structure around hydrophobic solutes and the Iceberg model. J. Phys. Chem. B.

[bib9] Gallagher L.R., Hartel R.W. (2025). Microstructural evolution of ice cream after start-up in a continuous scraped surface freezer. J. Food Eng..

[bib10] Gharsallaoui A., Rogé B., Génotelle J., Mathlouthi M. (2008). Relationships between hydration number, water activity and density of aqueous sugar solutions. Food Chem..

[bib11] Goff H.D., Hartel R.W. (2013).

[bib12] Gomes A., Bourbon A.I., Peixoto A.R., Silva A.S., Tasso A., Almeida C., Nobre C., Nunes C., Sánchez C., Gonçalves D.A., Castelo-Branco D., Figueira D., Coelho E., Gonçalves J., Teixeira J.A., Pastrana Castro L.M., Coimbra M.A., Pintado M., Ribeiro Cerqueira M.Â.P. (2023).

[bib13] Grembecka M. (2015). Sugar alcohols—Their role in the modern world of sweeteners: a review. Eur. Food Res. Technol..

[bib14] Güven M., Karaca O.B. (2002). The effects of varying sugar content and fruit concentration on the physical properties of vanilla and fruit ice-cream-type frozen yogurts. Int. J. Dairy Technol..

[bib15] Himashree P., Sengar A.S., Sunil C.K. (2022). Food thickening agents: sources, chemistry, properties and applications - a review. Int. J. Gastron. Food Sci..

[bib16] Hosseini S.M.S., Falamaki C. (2015). An efficient algorithm for modeling the thermodynamics of multi-solute adsorption from liquids. Fluid Phase Equilib..

[bib17] Hu R., Zhang M., Liu W., Mujumdar A.S., Bai B. (2022). Novel synergistic freezing methods and technologies for enhanced food product quality: a critical review. Compr. Rev. Food Sci. Food Saf..

[bib18] Jeong M., Gilmore J.S., Bleakley A., Jordan A. (2014). Local news media framing of obesity in the context of a sugar-sweetened beverage reduction media campaign. J. Nutr. Educ. Behav..

[bib19] Kawai K., Hagiwara T. (2018). Survival Strategies in Extreme Cold and Desiccation.

[bib20] Knopf D.A., Alpert P.A. (2013). A water activity based model of heterogeneous ice nucleation kinetics for freezing of water and aqueous solution droplets. Faraday Discuss..

[bib21] Kurultay Ş., Öksüz Ö., Gökçebağ Ö. (2010). The influence of different total solid, stabilizer and overrun levels in industrial ice cream production using coconut oil. J. Food Process. Preserv..

[bib22] Laage D., Elsaesser T., Hynes J.T. (2017). Water dynamics in the hydration shells of biomolecules. Chem. Rev..

[bib23] Lamas C.P., Vega C., Noya E.G. (2022). Freezing point depression of salt aqueous solutions using the Madrid-2019 model. J. Chem. Phys..

[bib24] Leighton A., Williams O.E. (1927). The basic viscosity of ice-cream mixes. J. Phys. Chem..

[bib25] Lerbret A., Bordat P., Affouard F., Descamps M., Migliardo F. (2005). How homogeneous are the trehalose, maltose, and sucrose water solutions? An insight from molecular dynamics simulations. J. Phys. Chem. B.

[bib26] Leyva-Porras C., Cruz-Alcantar P., Espinosa-Solís V., Martínez-Guerra E., Piñón-Balderrama C.I., Compean Martínez I., Saavedra-Leos M.Z. (2020). Application of differential scanning calorimetry (DSC) and modulated differential scanning calorimetry (MDSC) in food and drug industries. Polymers.

[bib27] Liu X., Sala G., Scholten E. (2023). Role of polysaccharide structure in the rheological, physical and sensory properties of low-fat ice cream. Curr. Res. Food Sci..

[bib28] López-Leiva M., Hallström B. (2003). The original plank equation and its use in the development of food freezing rate predictions. J. Food Eng..

[bib29] Maneffa A.J., Stenner R., Matharu A.S., Clark J.H., Matubayasi N., Shimizu S. (2017). Water activity in liquid food systems: a molecular scale interpretation. Food Chem..

[bib30] Marshall T., Marangoni A.G., Laredo T., Al-Abdul-Wahid M.S., Pensini E. (2022). Mechanisms of solvent separation using sugars and sugar alcohols. Colloids Surf. A Physicochem. Eng. Asp..

[bib31] McCain H.R., Kaliappan S., Drake M.A. (2018). Invited review: sugar reduction in dairy products. J. Dairy Sci..

[bib32] Mizan T.I., Savage P.E., Ziff R.M. (1996). Temperature dependence of hydrogen bonding in supercritical water. J. Phys. Chem..

[bib33] Mohammed M., Reiser P. (1995).

[bib34] Muse M.R., Hartel R.W. (2004). Ice cream structural elements that affect melting rate and hardness. J. Dairy Sci..

[bib35] Ni C., Gong Y., Liu X., Sun C.Q., Zhou Z. (2017). The anti-frozen attribute of sugar solutions. J. Mol. Liq..

[bib36] Ohtake S., Wang Y.J. (2011). Trehalose: current use and future applications. J. Pharmaceut. Sci..

[bib37] Park J., Ha M.Y., Min J.K. (2022). A numerical study on the effect of varying viscosity on the heat transfer inside pipes under low temperature conditions. J. Mech. Sci. Technol..

[bib38] Park S., Onufrak S., Sherry B., Blanck H.M. (2014). The relationship between health-related knowledge and sugar-sweetened beverage intake among US adults. J. Acad. Nutr. Diet..

[bib39] Penkov N.V. (2021). Relationships between molecular structure of carbohydrates and their dynamic hydration shells revealed by terahertz time-domain spectroscopy. Int. J. Mol. Sci..

[bib40] Penkov N.V. (2023). Terahertz spectroscopy as a method for investigation of hydration shells of biomolecules. Biophys. Rev..

[bib41] Płowaś-Korus I., Buchner R. (2019). Structure, molecular dynamics, and interactions in aqueous xylitol solutions. Phys. Chem. Chem. Phys..

[bib42] Portmann M.O., Serghat S., Mathlouthi M. (1992). Study of some factors affecting intensity/time characteristics of sweetness. Food Chem..

[bib43] Qian S., Hu F., Mehmood W., Li X., Zhang C., Blecker C. (2022). The rise of thawing drip: freezing rate effects on ice crystallization and myowater dynamics changes. Food Chem..

[bib44] Roy P., Menon S., Sengupta N. (2022). Dynamical manifestations of supercooling in amyloid hydration. J. Phys. Chem. B.

[bib45] Scholten E., Merkus H.G., Meesters G.M.H. (2014). Particulate Products: Tailoring Properties for Optimal Performance.

[bib46] Schultze-Jena A., Boon M.A., Vroon R.C., Bussmann P. J. Th, Janssen A.E.M., van der Padt A. (2020). High viscosity preparative chromatography for food applications. Separ. Purif. Technol..

[bib47] Shalaev E., Soper A., Zeitler J.A., Ohtake S., Roberts C.J., Pikal M.J., Wu K., Boldyreva E. (2019). Freezing of aqueous solutions and chemical stability of amorphous pharmaceuticals: water clusters hypothesis. J. Pharmaceut. Sci..

[bib48] Sloman B.M., Please C.P., Gorder R.A.V. (2020). Melting and dripping of a heated material with temperature-dependent viscosity in a thin vertical tube. J. Fluid Mech..

[bib49] Soukoulis C., Lebesi D., Tzia C. (2009). Enrichment of ice cream with dietary fibre: effects on rheological properties, ice crystallisation and glass transition phenomena. Food Chem..

[bib50] Starzak M., Peacock S.D., Mathlouthi M. (2000). Hydration number and water activity models for the sucrose-water system: a critical review. Crit. Rev. Food Sci. Nutr..

[bib51] Susrisweta B., Veselý L., Štůsek R., Hauptmann A., Loerting T., Heger D. (2023). Investigating freezing-induced acidity changes in citrate buffers. Int. J. Pharm..

[bib52] Suzuki T. (2007). The hydration of glucose: the local configurations in sugar–water hydrogen bonds. Phys. Chem. Chem. Phys..

[bib53] Tan M., Mei J., Xie J. (2021). The formation and control of ice crystal and its impact on the quality of frozen aquatic products: a review. Crystals.

[bib54] Tas O., Ertugrul U., Grunin L., Oztop M.H. (2022). Investigation of the hydration behavior of different sugars by time Domain-NMR. Foods.

[bib55] Telis V.R.N., Telis-Romero J., Mazzotti H.B., Gabas A.L. (2007).

[bib56] VanWees S.R., Rankin S.A., Hartel R.W. (2020). The microstructural, melting, rheological, and sensorial properties of high-overrun frozen desserts. J. Texture Stud..

[bib57] Vuist J.E., Schutyser M.A.I., Boom R.M. (2022). Solute inclusion during progressive freeze concentration: a state diagram approach. J. Food Eng..

[bib58] Wan Z., Khubber S., Dwivedi M., Misra N. (2021). Strategies for lowering the added sugar in yogurts. Food Chem..

[bib59] Wang H., Vanapalli S.K., Li X. (2025). A unified model for the soil freezing characteristic curve based on pore size distribution and principles of thermodynamics. Water Resour. Res..

[bib60] Wang R., Hartel R.W. (2020). Effects of moisture content and saccharide distribution on the stickiness of syrups. J. Food Eng..

[bib61] Whelan A.P., Regand A., Vega C., Kerry J.P., Goff H.D. (2008). Effect of trehalose on the glass transition and ice crystal growth in ice cream. Int. J. Food Sci. Technol..

[bib62] Whelan A.P., Vega C., Kerry J.P., Goff H.D. (2008). Physicochemical and sensory optimisation of a low glycemic index ice cream formulation. Int. J. Food Sci. Technol..

[bib63] Wu B., Freire D.O., Hartel R.W. (2019). The effect of overrun, fat destabilization, and ice cream mix viscosity on entire meltdown behavior. J. Food Sci..

[bib64] Wu D., Cao Y., Huang Q. (2023). Trehalose and sodium pyrophosphate inhibit ice-induced freezing quality deterioration of surimi: a comparative study on water migration, ice crystal growth, glass transition and state diagram. J. Food Eng..

[bib65] Yang Z., Xu D., Zhou H., Wu F., Xu X. (2022). New insight into the contribution of wheat starch and gluten to frozen dough bread quality. Food Biosci..

[bib66] Yuennan P., Sajjaanantakul T., Goff H.D. (2014). Effect of okra cell wall and polysaccharide on physical properties and stability of ice cream. J. Food Sci..

[bib67] Zhu Z., Zhou Q., Sun D.-W. (2019). Measuring and controlling ice crystallization in frozen foods: a review of recent developments. Trends Food Sci. Technol..

[bib68] Zuorro A. (2021). Water activity prediction in sugar and polyol systems using theoretical molecular descriptors. Int. J. Mol. Sci..

